# The Hidden Challenge: Pelvic Floor Symptoms and Their Impact on Performance and Well‐Being in Elite Female Rugby Players

**DOI:** 10.1002/ejsc.70013

**Published:** 2025-07-12

**Authors:** Jodie Dakic, Luke Perraton, Jessica Lindstrom, Elana Hain, Shinyi Chuah, Sharon Stay

**Affiliations:** ^1^ Department of Physiotherapy Monash University Frankston Australia; ^2^ National Tennis Academy Tennyson Australia; ^3^ Queensland Rugby Union Herston Australia

**Keywords:** incontinence, pelvic floor, pelvic pain, rugby, sport

## Abstract

More than half of female ball‐sport athletes experience urinary leakage including 60% of Australian rugby union players. Beyond urinary incontinence (UI), the prevalence and impact of other pelvic floor (PF) symptoms in elite female athletes remains largely unknown. This study investigated PF symptom prevalence and impact on performance in elite female rugby players. A cross‐sectional observational study was conducted. Australian elite female rugby players (*n* = 56) completed a PF questionnaire during annual medical screening. Validated questionnaires established: presence/severity of UI (ICIQ‐UI SF), presence and bother of PF symptoms (PFBQ), urinary tract infection, constipation, voiding difficulty (APFQ) and pelvic pain (adapted UDI‐6). Purpose‐designed questions established symptom impact on performance and well‐being. Data were analysed descriptively. Response/questionnaire completion rates were > 95%. Participants were on average: 22.4 years (SD: 5.9); BMI 26.3 kg/m^2^ (SD: 4.6); mostly nulliparous (94%) and played 6.5 years of rugby (SD: 4.4). More than half (57%) experienced PF symptoms during sport, most commonly UI (41%). Other PF symptoms experienced during sport included: AI (29%), bladder urgency (21%)/frequency (14%) and pelvic pain (12%). One in two symptomatic players reported an impact on performance (53%) including training reductions (34%); activity avoidance (25%) and concentration loss (9%). Players restricted fluid intake to avoid provoking symptoms. A quarter of players reported PF symptoms that impacted their sports performance. This is the first study to establish that, beyond UI, AI, bladder urgency/frequency and pelvic pain are also prevalent in elite rugby players. Elite female rugby players require assessment and management of PF symptoms to maximise performance and well‐being.

## Summary

- One in two elite female rugby players experienced pelvic floor symptoms during training or competition, most commonly urinary and anal incontinence, bladder frequency/urgency and pelvic pain.
- Half of the symptomatic players experienced an impact of PF symptoms on their performance causing modification to training load or activities and/or a loss of focus during sport.
- Players used a variety of coping and management strategies including emptying their bladder frequently, restricting fluid intake and preferring uniforms that allow symptom concealment.

## 1 Introduction

Women's rugby is growing in popularity and participation (McLachlan 2019). Rugby is a physically demanding team sport requiring players to participate in intermittent efforts of jogging, sprinting, tackling and scrummaging. Players cover large distances running (high and low speed) and experience multiple body contacts per game (Suarez‐Arrones et al. 2014).

Pelvic health is an important yet under recognised aspect of women's rugby. The pelvic floor (PF) is comprised of passive tissues (ligaments, fascia and pelvic organs) and active tissues (PF muscles and their nerve supply) (Bo et al. 2017). During sport or exercise, exertion causes a rise in intrabdominal pressure (IAP) (Dietze‐Hermosa et al. 2020). In high‐impact sports involving running or jumping, ground reaction forces (GRF) are generated, and loads at least three times body weight are transmitted (Bø and Nygaard 2020). Rises in IAP and GRF are transferred down through the PF and must be countered by the active and passive PF tissues (Bø and Nygaard 2020). Sports involving high impact activities, body contact and heavy lifting may place the PF under substantial load. During a rugby game, a player is repetitively exposed to numerous loads from direct contact to the torso (e.g., tackle) or shoulders (e.g., scrum) or via gravity when running or landing. These loads may cause rises in IAP and GRF that will be transferred through the PF. (Donnelly, Bø et al. 2024). The effect these loads will have on PF function will vary between individuals and may change over time. Any change to the structure or function of the PF can lead to a group of conditions called PF disorders (Messelink et al. 2005).

Symptoms of PF disorders such as urinary incontinence (UI) are prevalent in female athletes (Teixeira et al. 2018). One in three female athletes and up to 80% of those playing high‐impact sports experience leaking urine (de Mattos Lourenco et al. 2018). In rugby, the prevalence of UI was studied via an online social media survey. In a large cohort of UK and Irish rugby players, across all levels of participation (*n* = 396), 63% reported UI with 43% experiencing symptoms whilst playing rugby (McCarthy‐Ryan et al. 2024). One in three players reported modifying their participation in rugby because of their symptoms (McCarthy‐Ryan et al. 2024). Urinary incontinence prevalence was also investigated in Australian rugby union players (Faulks and Catto 2021). 60 percent had experienced UI during rugby, although, with a low response rate to the questionnaire (12%) it is difficult to draw conclusions on the true prevalence in the elite population (Faulks and Catto 2021).

Beyond UI, there is scarce literature on the prevalence of other PF symptoms experienced by female rugby players. A study of three varsity rugby teams in Alberta, Canada, reported a prevalence of UI (54%), urinary urgency (53%)/frequency (39%) and pelvic pain in the lower abdominal, pelvic or genital area (26%) (Sandwith and Robert 2021). Anal incontinence (AI) (accidental leakage of wind or stool) has been found to be prevalent in other elite female athletes such as power/weight lifters and gymnasts (approximately 80%) (Skaug et al. 2022a, 2022b). Both UI and AI occur at higher rates in elite athletes than age matched controls (Carvalhais et al. 2018; Carvalho et al. 2020). In a large observational study of community dwelling women, playing varying sports, 17% reported symptoms of pelvic organ prolapse (POP) (Campbell et al. 2023). The prevalence of PF symptoms in elite rugby players requires exploration to establish the diverse range of PF symptoms that may be experienced in this demanding sport. The impact of these symptoms on performance, emotional and social well‐being and the strategies elite rugby players use to cope/manage symptoms also requires investigation. A recent expert review called for research focusing on pelvic health in rugby players in order to define the problem and inform surveillance, education and management strategies that may be required within sports settings. (Donnelly, Bø et al. 2024). The aim of this study was to investigate the prevalence of PF symptoms and their impact on performance and well‐being in Australian elite female rugby players.

## 2 Materials and Methods

A cross‐sectional observational study investigating the prevalence and impact of PF symptoms was conducted. The study was approved by a Human Ethics Research Committee (project number: 34414, 29/11/2023). The Strengthening the Reporting of Observational Studies in Epidemiology (STROBE) (von Elm et al. 2008) checklist was used for reporting.

### 2.1 Participants

The participants were Australian elite female rugby players. Super rugby women's players represented Queensland (QLD) at a National level of Competition. Rugby sevens players represented their state (QLD) in national level competition; participants from both groups also represented Australia or were in national team selection pathways.

### 2.2 Data Collection

A written paper questionnaire was used to collect data administered in‐person by the Queensland rugby union Medical Officer (SS) at their training facility. Pelvic health screening was offered to players as part of Rugby Australia's annual medical, cardiac and concussion screening (March 2023). Players were informed that they had the option to consent for their data to be used for future research processes and were provided with a written consent form. They were informed that consent was voluntary, and they could still participate in the medical screening process if they did not consent to their data being used for research. Consent was also provided by a representative from Rugby Australia.

The questionnaire was purpose‐designed using validated questionnaires when available. Participant characteristics were collected: age, body mass index (BMI), sports played and level of participation, number of years played, medical co‐morbidities and menstrual and menopausal status. Obstetric history and birth risk factors for the development of PF disorders were also collected. The time to return to sport (training, competition and pre‐childbirth level of participation) after birth were recorded.

The International Consultation on Incontinence Questionnaire—Urinary Incontinence Short Form (ICIQ‐UI SF) (Abrams et al. 2006) was used to assess for the presence of UI symptoms and their impact on quality of life as recommended by the International Consensus on Incontinence (ICI) 2023 (Castro Diaz et al. 2023). The ICIQ‐UI SF has been deemed to have content, concurrent and discriminant validity and reliability in women (Castro Diaz et al. 2023). It contains four questions determining the frequency (‘never’ to ‘all of the time’) and volume (‘none’ to ‘a large amount’) of UI; the situations that provoke urinary leakage and the amount leakage interferes with everyday life (scale of 0 [not at all] to 10 [a great deal]) (Abrams et al. 2006). Athletes were deemed continent if they answered ‘never’ to the question: ‘How often do you leak urine?’ Urinary incontinence was classified into sub‐types using the response to question four: ‘When does urine leak?’ Participants who responded affirmatively to ‘leaks when you cough or sneeze’ and/or ‘leaks when you are physically active/exercising’ were categorised as experiencing stress urinary incontinence (SUI); participants who responded affirmatively to ‘leaks before you can get to the toilet’ were categorised as experiencing urgency urinary incontinence (UUI) and participants who reported both SUI and UUI were categorised as experiencing mixed urinary incontinence (MUI). The degree of bother of UI symptoms was determined by using a question from the pelvic floor bother questionnaire (PFBQ) (Peterson et al. 2010): ‘How much does leaking urine bother you’, with responses ranging from ‘not at all’ to ‘a lot’.

The presence and degree of bother of other PF symptoms were determined through use of the PFBQ (Peterson et al. 2010). The PFBQ contains nine questions related to symptoms of urinary urgency and frequency, dysuria, POP, obstructed defecation, AI and dyspareunia. Presence of symptoms was identified through a response of ‘yes’ and degree of bother using a scale (‘not at all’; ‘only a little bit’; ‘somewhat’; ‘a moderate amount’ or ‘a lot’) (Peterson et al. 2010). In order to reduce participant burden, two questions used to identify UI sub‐type were excluded as UI sub‐type had already been established with the ICIQ‐UI SF. The PFBQ has good correlation with the pelvic floor impact questionnaire and the pelvic floor distress inventory (Peterson et al. 2010). It is recommended for use in clinical settings to identify a range of symptoms and was selected as the primary purpose of the pelvic health questionnaire was inclusion in annual medical screening. It has good reliability and good to very good correlation with objective measures of POP (measured by POP‐Q) and bowel/bladder clinical diagnosis (measured by urodynamics and defecography) (Peterson et al. 2010).

A question to identify the presence of pelvic pain was included. There are currently no concise questions to identify the presence or bother of pelvic pain validated in female athletes. A question from the Urogenital Distress Inventory (UDI‐6) (van der Vaart et al. 2003; Shumaker et al. 1994), ‘Do you experience pain or discomfort in your lower abdominal, pelvic or genital area?’, was selected. We added a further question to collect details on perceived location of pain by using language that aligns with common complaints of pain symptoms from the Standard Terminology by the Chronic Pelvic Pain Working Group of the International Continence Society (Doggweiler et al. 2017). We included the question ‘Do you experience pain or discomfort in your lower abdominal, pelvic or genital area?’ and offered responses: ‘in your bladder (which may include pain with holding on to or emptying urine)’; ‘in your bowel (which may include pain with holding on to stool or emptying stool)’; ‘in your vagina (which may include pain with use of tampons)’ or ‘in your uterus (which may include period pain)’.

Three questions from the validated Australian Pelvic Floor Questionnaire (AFPQ) (Baessler et al. 2009) for routine clinical screening and research were included to evaluate possible risk factors for PF disorders (UI, AI and POP): straining on the toilet and recurrent urinary tract infections (UTI). The language was modified slightly to align with questions used in previous research on PF symptom risk factors in elite female athletes to allow comparison of findings (Skaug et al. 2022a, 2022b). Straining on the toilet was assessed using ‘Do you need to strain to open your bowels?’ and ‘Do you need to strain to empty your bladder?’ A question to identify recurrent UTI's was included: ‘Do you experience frequent bladder infections?’

Participants were then asked the types of PF symptoms they experienced whilst exercising and which sporting activities provoked symptoms. These questions have been previously piloted and used in other research on PF symptom impact in exercising women (Dakic et al. 2022, 2021). The impact of PF symptoms on performance, mental and social well‐being were assessed using the question ‘Do you think your pelvic floor symptoms negatively impact your performance in any of the following ways?’ and ‘Do you experience any of the following due to your pelvic floor symptoms?’ Participants were also asked about the coping strategies they used to manage their symptoms. Responses were developed using previous research on PF disorders in female athletes (Culleton‐Quinn et al. 2022; Dakic, Hay‐Smith, Lin, Cook, and Frawley 2023b). An open free‐text response box was provided.

The degree of impact on performance and how much the impact bothered participants was also assessed using a five‐point scale from ‘not at all’ to ‘a lot/greatly’.

### 2.3 Statistical Analysis

Chi‐squared analysis for categorical data and *t*‐test for continuous data were used to compare demographics between Rugby sevens and Super Rugby Women's participants. One (*n* = 1) participant was removed from sub‐group analysis as they reported playing both leagues but their data were included in the whole cohort analysis. To preserve participant anonymity, we did not report obstetric data (beyond parous [yes/no]) as responses to all variables were *n* < 5. Data were reported descriptively. Chi squared analysis was used to compare PF symptom prevalence between Rugby sevens and Super Rugby Women's players. A value of *p* ≤ 0.05 determined statistical significance. Data analysis was performed using statistical software (SPSS Statistics for Windows, Version 28. Armonk, NY:IBM Corp.) Where answers were illegible on the written survey, they were removed from the analysis for that question and reported as missing data in results tables. Missing data were also reported if a participant chose not to answer a question. For example, *n* = 6 participants chose not to answer the question on pain during sexual activity (responses were not forced). For questions on activities that provoke symptoms, coping strategies and impacts on performance/well‐being, free‐text responses were allowed. Responses were categorised and frequency counted.

## 3 Results

All members of the state (QLD) Rugby sevens and Super Rugby Women's extended squads attended annual medical screenings as part of the World Rugby requirement for annual medical assessment. All participants were offered the opportunity to participate in the study. Of those, 100% of Rugby sevens players (*n* = 27) and 91% of Super Rugby Women's players (*n* = 29 of 32) consented to participate in the study. Fifty‐six participants completed 100% of the questionnaire.

Participants had a mean age of 22.4 years (±5.9, range: 15–36 years) and mean BMI: 26.3 (±4.6) (kg/m^2^) (Table 1). On average participants had played in their sport league for 6.5 years (±4.4) and at the elite level for 3.3 years (±2.3). Rugby sevens participants were significantly (*p* < 0.001) younger (mean:17.9 vs. 26.4 years respectively) and had lower BMI (mean: 24.0 vs. 28.1 kg/m^2^ respectively) than Super Rugby Women's participants. Most participants (95%. *n* = 53) were nulliparous; three Super Rugby Women's players had experienced childbirth.

**TABLE 1:** Participant characteristics (*n* = 56); [rugby 7's (*n* = 27), super rugby women's (*n* = 28)].

Characteristics	Overall (*n* = 56)^a^ mean (SD)	Rugby sevens (*n* = 27) mean (SD)	Super rugby women's (*n* = 28) mean (SD)
Socio‐demographics			
Age (years)	22.4 (± 5.9) range:15 to 36	17.9 (± 2.4) range:15 to 24	26.4 (± 5.0) range:19 to 36
BMI (kg/m^2^)	26.3 (± 4.6)	24.0 (± 2.7)	28.1 (± 5.1)
Sport participation history			
Number of years playing primary sport (years)	6.5 (± 4.4)	5.6 (± 3.4)	6.9 (± 4.4)
Number of years playing at elite level (years)	3.3 (± 2.3)	2.7 (± 1.5)	3.6 (± 2.2)
Urogynecology variables			
Age of first period (years) (mean [SD])	13.2 (± 1.7)	13.0 (± 1.4)	13.4 (± 2.0)
Menopause (yes)	0	0	0

Obstetric variables	*n* (%)	*n* (%)	*n* (%)
Parity			
Nulliparous	53 (94.6)	27 (100)	25 (89.3)
Parous	3 (3.6)	0 (0)	3 (10.7)

Abbreviations: BMI, body mass index; SD, standard deviation.

^a^ *n* = 1 removed from rugby type analysis as played both Rugby sevens and Super Rugby Women's.

The most prevalent PF symptoms reported were straining to empty the bowel (46%, *n* = 26) and UI (41%, *n* = 23) (Table 2). One in four participants experienced SUI (*n* = 15) and 7% UUI or MUI. A majority of participants with UI reported mild symptoms (73%) and 21% reported moderate severity. One in four participants reported urinary frequency (*n* = 15) or urgency (*n* = 13). One in five participants reported pelvic pain and/or dyspareunia (pain during sexual activity); half of those with pelvic pain found it to be more than a little bit bothersome. The prevalence of POP symptoms (4%, *n* = 2) and AI (2%, *n* = 1) measured by PFBQ were low. There was no significant difference (*p* < 0.05) in prevalence of PF symptoms between Rugby sevens and Super Rugby Women's players.

**TABLE 2:** Pelvic floor symptom: prevalence, severity, frequency and bother (*n* = 56).

Pelvic floor disorder type	Prevalence *n* (%)			
	Overall (*n* = 56)	Rugby sevens (*n* = 27)	Super rugby women's (*n* = 28)	*p*‐value
Straining to empty bowel	26 (46.4)	10 (37.0)	15 (53.6)	0.34
Frequency				
Never	30 (53.6)	17 (63.0)	13 (46.4)	
Rarely	16 (28.6)	6 (22.2)	9 (32.1)	
Some of the time	10 (17.9)	4 (14.8)	6 (21.4)	
Most of the time	0	0	0	
Always	0	0	0	
Urinary incontinence (UI)	23 (41.1)	10 (37.0)	13 (46.4)	0.67
UI sub‐type				
SUI	15 (26.8)	7 (25.9)	8 (28.6)	1.0
UUI	4 (7.1)	2 (7.4)	2 (7.1)	1.0
MUI	4 (7.1)	1 (3.7)	3 (10.7)	0.61
Severity^a^				
Mild	17 (35.7)			
Moderate	5 (8.9)			
Severe	0			
Very severe	0			
Bother (*n* = 23)				
Not at all	8			
Only a little bit	12 (21.4)			
Somewhat	2 (3.6)			
A moderate amount	1 (1.8)			
A lot	0			
Urinary frequency	15 (26.8)	6 (22.2)	9 (32.1)	0.60
Bother				
Not at all	1 (1.8)			
Only a little bit	6 (10.7)			
Somewhat	3 (5.4)			
A moderate amount	2 (3.6)			
A lot	1 (1.8)			
Missing	2 (3.6)			
Urinary urgency	13 (23.2)	4 (14.8)	8 (28.6)	0.33
Bother				
Not at all	0			
Only a little bit	7 (12.5)			
Somewhat	5 (8.9)			
A moderate amount	1 (1.8)			
A lot	0			
Lower abdominal, pelvic or genital pain	11 (19.6)	4 (14.8)	6 (21.4)	0.73
Location of pelvic pain^b^				
Bladder pain	2 (3.6)			
Bowel pain	2 (3.6)			
Vagina pain	5 (8.9)			
Uterine pain	8 (14.3)			
Bother				
Not at all	0			
Only a little bit	6 (10.7)			
Somewhat	1 (1.8)			
A moderate amount	3 (5.4)			
A lot	1 (1.8)			
Pain impacting sexual intercourse (*n* = 37)^c^	8 (21.6)	3 (27.3)	4 (16.0)	0.66
Bother				
Not at all	23 (62.2)			
Only a little bit	6 (16.2)			
Somewhat	1 (2.7)			
A moderate amount	1 (2.7)			
A lot	0			
Missing	6			
Recurrent urinary tract infections	10 (17.9)	3 (11.1)	7 (25.9)	0.29
Frequency				
Never	45 (80.4)	24 (88.9)	20 (74.1)	
1–3 times per year	10 (17.9)	3 (11.1)	7 (25.9)	
4–12 times per year	0	0	0	
More than once per month	0	0	0	
Missing	1			
Straining to empty the bladder	7 (12.5)	4 (14.8)	3 (10.7)	0.71
Frequency				
Never	49 (87.5)	23 (85.2)	25 (89.3)	
Occasionally	7 (12.5)	4 (14.8)	3 (10.7)	
Frequently	0	0	0	
Daily	0	0	0	
Difficulty or discomfort emptying bowel	6 (10.7)	2 (7.4)	4 (14.3)	0.67
Bother				
Not at all	0			
Only a little bit	6 (10.7)			
Somewhat	0			
A moderate amount	0			
A lot	0			
Pelvic organ prolapse	2 (3.6)	1 (3.7)	1 (3.6)	1.0
Bother				
Not at all	0			
Only a little bit	1 (1.8)			
Somewhat	1 (1.8)			
A moderate amount	0			
A lot	0			
Anal incontinence	1 (1.8)	1 (3.7)	0	0.49
AI bother				
Not at all	1 (1.8)			
Only a little bit	0			
Somewhat	0			
A moderate amount	0			
A lot	0			
Difficulty or discomfort urinating	0	0	0	0

*Note:* Where a category value for prevalence is < 5 Fischer's Exact Probability Test is reported for *p*.

^a^ *n* = 2 missing for UI severity due to responses that could not be interpreted.

^b^ Participants could select multiple responses to pain location.

^c^ % of those who reported being sexually active.

More than half (57%, *n* = 32) of the participants had experienced PF symptoms during sport or exercise activities. The most commonly experienced symptom was urinary leakage (41%) followed by AI (29%), bladder urgency (21%) and frequency (14%) and pelvic pain (12%) (Table 3). Approximately half (*n* = 14) of those with symptoms experienced them during competition (43.8%, *n* = 14). Symptoms were common during tackling (50%), jumping (31%), lifting weights (19%) and practising new skills (16%). One in four experienced symptoms in the first half of training.

**TABLE 3:** Pelvic floor symptoms experienced during sport (*n* = 56), impact on performance and degree of impact and bother on performance.

	Prevalence *n* (%)
Pelvic floor symptoms experienced whilst playing sport or exercising (*n* = 56)	
Accidently leaked urine	23 (41.1)
Accidently passed wind	16 (28.6)
Needed to get to the toilet in a hurry or not made it there on time	12 (21.4)
Constantly needed to go to the toilet	8 (14.3)
Pain in the bladder, bowel, vagina or uterus	7 (12.5)
Experienced a bulging sensation, wind or heaviness in the vagina	1 (1.8)
Lost control of bowels/accidental leakage of stool	0
When are pelvic floor symptoms experienced (*n* = 32)^a^	
Tackling	16 (50.0)
Competition	14 (43.8)
Jumping	10 (31.3)
First half of training	9 (28.1)
Second half of training	6 (18.8)
Lifting weights	6 (18.8)
Learning new activities or exercises	5 (15.6)
Landing	5 (15.6)
Jogging	4 (12.5)
Sprinting	4 (12.5)
Other	1 (3.1)
Impact of symptoms on performance (*n* = 32)^a^	
Reduced intensity during training	11 (34.4)
Avoidance of certain exercises/skills, for example, jumping	8 (25)
Loss of concentration	6 (18.8)
Distraction leading to mistakes	3 (9.4)
Avoid exercising in group settings or with other people	3 (9.4)
Change to a low‐impact version of more high‐impact exercise/activity	2 (6.3)
Reduced intensity during competition	1 (3.1)
Other	1 (3.1)
Degree of impact of symptoms on performance (*n* = 17)^b^	
Not at all	3 (17.6)
Only a little bit	9 (52.9)
Somewhat	1 (5.9)
A moderate amount	1 (5.9)
A lot	0
Missing (*n* = 3)	
Level of bother because of symptom impact on performance (*n* = 17) ^b^	
Not at all	3 (17.6)
Somewhat	9 (52.0)
Moderately	2 (11.8)
Greatly	0
Missing (*n* = 3)	

^a^ % reported of those who experienced PF symptoms during sport (*n* = 32), participants could select multiple responses.

^b^ % reported of those who reported an impact of PF symptoms on performance (*n* = 17).

One in two symptomatic players reported that their PF symptoms impacted their performance (53%, *n* = 17). Impacts included: reduced training intensity (34%); activity avoidance (e.g., not jumping) (25%) and loss of concentration during sport (9%). Ten percent avoided playing sport or exercising around others where possible. One in three symptomatic players (34%, *n* = 11) were either somewhat or moderately bothered by the impact of PF symptoms on their performance.

Players with symptoms emptied their bladder before training (81%), restricted their fluid intake before training or during competition (25%) and wore dark coloured clothing (19%) or a pad (19%) to reduce or manage symptoms (Table 4). One in three symptomatic participants (34%, *n* = 11) were somewhat/moderately bothered by the strategies they used to cope with or manage their symptoms. Of those participants who had not experienced PF symptoms during sport, one in three (*n* = 9) emptied their bladder before training or competing. Six of these participants had experienced PF symptoms during activities of daily living but not during sport.

**TABLE 4:** Management and coping strategies for pelvic floor symptoms during sport or exercise.

Method for coping or managing symptoms during sport or exercise^a^	Total cohort (*n* = 56) *n* (%)	Those that experience PF symptoms during sport (*n* = 32) *n* (%)	Those that do *not* experience PF symptoms during sport (*n* = 24) *n* (%)
Empty bladder before training and competition	35 (62.5)	26 (81.3)	9 (37.5)
Limiting fluid intake before competition and training	9 (16.1)	8 (25.0)	1 (4.2)
Use containment product during sport (e.g., pad)	6 (10.7)	6 (18.8)	0
Wear darker or different clothing	6 (10.7)	6 (18.8)	0
Empty bladder during training and competition	5 (8.9)	3 (9.4)	2 (8.3)
Limiting fluid intake during competition and training	4 (7.1)	3 (9.4)	1 (4.2)
Use tampon during competition or training	1 (1.8)	1 (3.1)	0
No coping or management strategies used	19 (33.9)	4 (12.5)	15 (62.5)
Total using coping mechanisms	37 (66.1)	28 (87.5)	9 (37.5)

^a^ Participants could select multiple strategies.

**FIGURE 1:**
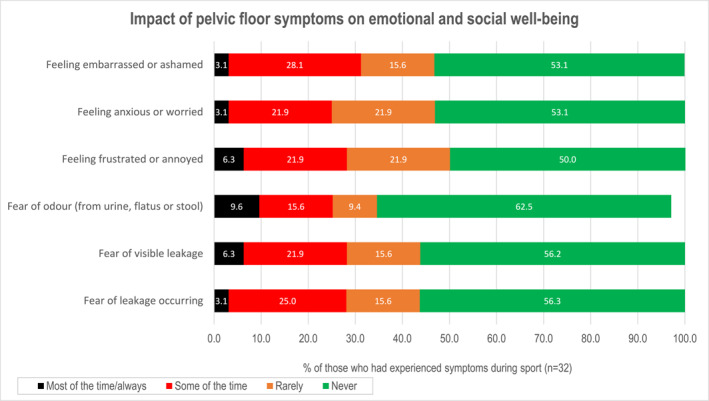
Impact of pelvic floor symptoms on emotional and social well‐being in elite female rugby players experiencing pelvic floor symptoms (*n* = 32). Fear of odour: missing data *n* = 1 (illegible response).

One in two symptomatic participants reported feeling embarrassed (47%), anxious or worried (47%) or frustrated/annoyed (50%) because of their PF symptoms. One in three were fearful of the odour of urine, flatus or stool; this concerned 10% of participants most/all of the time. Almost half of participants were fearful that leakage would become visible (Figure 1).

## 4 Discussion

One in two elite rugby players experienced PF symptoms during sport; of those with symptoms, half reported an impact on their performance including reducing or changing training to avoid provoking symptoms. This is the first study to establish that, in addition to UI, AI, bladder urgency/frequency and pelvic pain are also prevalent in elite female rugby players.

Urinary incontinence was the most prevalent symptom experienced by participants during sport (41%). Previous cross‐sectional survey studies have investigated prevalence of UI in rugby. Faulks, Catto (Faulks and Catto 2021) conducted a study of UI prevalence in elite Australian female rugby union participants. Sixty percent of participants reported UI during rugby, a higher prevalence than our findings (Faulks and Catto 2021). However, a low response rate of 12% (Faulks and Catto 2021) suggests a potential self‐selection bias as those players with symptoms may have deemed the survey of greater relevance. In the present study, across two rugby squads, 95% consented and fully completed the questionnaire. Therefore, the prevalence in our study is likely representative of the population of elite female rugby players.

Pregnancy or childbirth are inciting factors for the development of UI because of the trauma and load placed on the PF muscles and passive structures in the perinatal period (MacLennan et al. 2000). In addition, time away from competition and training during pregnancy and the early‐postnatal period may contribute to sport‐specific deconditioning, impacting the load tolerance of the PF and potentially further increasing the risk of PF symptom development (Donnelly, Coltman et al. 2024). Childbirth has been previously identified as a risk factor for experiencing UI during rugby (McCarthy‐Ryan et al. 2024) and it would be expected that nulliparous athletes, without a history of birth trauma, may have lower prevalence of UI. In a large study of female rugby players (*n* = 395) across the UK and Ireland, participating at any level, more than half of participants were parous (62%) (McCarthy‐Ryan et al. 2024). Despite a much higher level of parity in the UK and Irish cohort, we found similar prevalence in our group who were mostly nulliparous (McCarthy‐Ryan et al. 2024). A recent systematic review in high‐performing, adolescent female athletes reported UI prevalence at one in two (Rebullido et al. 2021). The reasons that nulliparous athletes, who have not experienced birth trauma, have such high prevalence of UI requires investigation.

The prevalence of PF symptoms in female athletes, beyond UI, has not been well investigated in elite sports, inclusive of rugby. Anal incontinence (accidental leakage of wind/stool) is experienced by 80% of elite power/weight lifters and gymnasts/cheerleaders (Skaug et al. 2022a, 2022b). Our findings indicated a much lower prevalence in elite rugby players; only 2% reported symptoms of AI when asked through a validated questionnaire if in the last month they had ‘experienced accidental leakage of faecal matter or gas (stool or wind)?’ However, when asked specifically if they had ever accidently leaked wind during sport or exercise, one in four players reported symptoms. It may be that symptoms of AI in this cohort are infrequent, or that it is important, when asking athletes about PF symptoms, to provide context with sport as a reference for when symptoms may be experienced.

There are scarce data on the prevalence of pelvic pain experienced during sport/exercise, particularly in elite populations. One in three elite endurance/cross country skiers reported pain in the lower abdominal or genital region, however, the specific site of pain or whether it was experienced during sport was not reported (Poświata et al. 2014). In a study of varsity level rugby players (*n* = 109), 26% reported pain in the lower abdominal, pelvic or genital region (Sandwith and Robert 2021). To the best of our knowledge, this is the first study to report on the prevalence of pelvic pain during sport in elite team sport athletes. Twenty percent had experienced pain in the lower abdominal, pelvic or genital region, a similar prevalence to varsity level players. Uterine pain was the most common perceived site of pelvic pain (14%). One in 10 players had experienced pain during rugby. One in four participants in our study who were sexually active had experienced dyspareunia (pain impacting sexual activity). The prevalence of dyspareunia has been previously reported as high as one in two female athletes, including in CrossFit practitioners (48.7%) (Pisani et al. 2021), cheerleaders (53.8%) (Carvalho et al. 2020) and professional dancers (44.8%) (Winder et al. 2023). We found low prevalence of POP symptoms in elite rugby players (4%). Pregnancy and vaginal birth are key risk factors for the development of POP. Previous studies have found the prevalence of POP to be higher in parous athletes (Gill et al. 2017; Yi et al. 2016). Participants in our study were mostly nulliparous, and so low POP prevalence was expected.

The reason that elite athletes experience a high prevalence of PF symptoms is currently not well understood. Typically, the skeletal muscles of athletes respond to the loads placed upon them by increasing in cross‐sectional area, strength, endurance and coordination. The effect of heavy and strenuous load on the PF and associated muscles is unknown. Bo and Nygaard (Bø and Nygaard 2020) have proposed two main theories: (i) elite sport may have a similar ‘training effect’ on the PF muscles as observed in other skeletal muscles, leading to improvement in strength, endurance and coordination or (ii) strenuous exertion may overload and weaken the PF tissue, leading to lack of structural support for the pelvic organs and the provocation of PF symptoms (Bø and Nygaard 2019). In rugby, there are a number of demands on the PF. The high incidence of body contact through the abdomen and shoulders from tackling and scrummaging influences the IAP, transferring forces to the PF. (Donnelly, Bø et al. 2024). Repeated sprinting efforts, acceleration/decelerations and jumping/landing create both IAP and GRFs which also require counter pressure from the PF. (Donnelly, Bø et al. 2024). Experiencing PF symptoms during rugby may indicate that a player's PF muscles and passive structures are unable to match the load placed upon them. (Donnelly, Bø et al. 2024). There are a number of factors that may contribute to insufficient PF function in elite female athletes. Many morphological factors have been investigated including: PF muscle cross‐sectional area (Kruger et al. 2005, 2007), squeeze pressure (Kruger et al. 2007; de Melo Silva et al. 2020; Bérubé and McLean 2024), coordination/timing (Moser et al. 2018; Koenig et al. 2020), hypermobility of passive structures, for example, urethral or bladder neck mobility (Kruger et al. 2007; Bérubé and McLean 2023, 2024), and anatomical considerations (e.g., levator hiatus (Bérubé and McLean 2023, 2024) or urogenital hiatus width (Kruger et al. 2005)). At this stage, there has been no conclusive evidence on the primary contributors to PF symptoms in nulliparous female athletes, and it may be that it is highly individualised.

Like others, we found that tackling, jumping and landing commonly provoked PF symptoms (McCarthy‐Ryan et al. 2024; Faulks and Catto 2021). The different codes of rugby place different demands on the individual. Super Rugby Women's is a national professional 15's rugby union competition in Australia governed by Rugby Australia. Rugby sevens is an alternative code of rugby union; there are fewer players than in a Super Rugby Women's rugby union game (seven instead of fifteen on a full pitch) and shorter halves (7 vs. 40 min). Rugby sevens players typically cover greater running distances than in a 15's match and Super Rugby Women's players typically experience more scrummages, mauling and high contact tackles (hundreds per game) (Paul et al. 2022). We hypothesised there may be a difference in PF symptom prevalence between rugby codes because of differing loads on PF tissues, that is, longer endurance/repeated interval running (Rugby sevens) versus greater numbers of body contact to the torso creating higher IAP (Super Rugby Women's). However, sub‐group analysis revealed no statistically significant difference in the prevalence of PF symptoms between rugby codes; a larger cohort may be required for further analysis.

Players attempted to reduce symptom provocation by modifying the way they trained and competed. They reduced the intensity of their training and avoided certain exercises such as jumping high or sprinting at full intensity. One in 10 had made mistakes as they were distracted by their PF symptoms. In a large cohort of UK and Irish rugby players, one in three modified their participation by changing their body position during contact activities or reducing the amount they lifted in weight training (McCarthy‐Ryan et al. 2024). For elite athletes, a reduction in the ability to train at full intensity or having to adapt to less than ideal techniques during tacking could result in reduced performance and potential injury risk. One in two symptomatic players in our study reported that their symptoms impacted their performance. In a previous study of elite Australian rugby players, 2 of 39 symptomatic players felt their PF symptoms were a barrier to continuing to play in the future (Faulks and Catto 2021). This study adds weight to a prior review of pelvic health in rugby by an expert panel, who advocated for the importance of screening and management of PF symptoms in rugby. (Donnelly, Bø et al. 2024). Early identification and treatment for PF symptoms may reduce symptom impact on performance and prevent players stopping rugby participation due to symptoms.

The PF muscles can be strengthened via specific exercises targeting their strength, endurance, coordination and ability to relax (Dumoulin et al. 2018). Pelvic floor muscle training is highly effective in community dwelling women for preventing and reducing UI, and reducing symptoms of AI and POP (Dumoulin et al. 2023). Non‐pharmacological conservative therapy has also been shown to be effective in managing chronic pelvic pain (Starzec‐Proserpio et al. 2024). Whilst studies on conservative management of PF symptoms in female athletes are scarce, preliminary results on the effect of PF muscle training to reduce stress UI are promising (Da Roza et al. 2012; Bø et al. 2024; Ferreira et al. 2014; Neels et al. 2017; Rivalta et al. 2010; Giagio et al. 2022; Fukuda et al. 2022; Romero‐Franco et al. 2021). Larger, more robust studies are imperative and the feasibility/acceptability of delivering pelvic health screening and management within elite sport settings also requires investigation.

The strengths of this study are the high response and completion rate amongst a cohort of elite female athletes. Although this study included players from only one Australian state, it is likely an accurate representation of prevalence in this population compared with prior studies with low response rates (Faulks and Catto 2021) or possible self‐selection bias (McCarthy‐Ryan et al. 2024). In addition, where available we used validated questionnaires and investigated a broad range of PF symptoms, many of which have not previously been investigated in elite team sport athletes. In person screening, embedded within injury and general health surveillance, allowed participants to clarify language and checking of response completion by a person familiar to the participants. Prior research by our group found that women recommended pelvic health screening questions be embedded within general health screening to help normalise PF symptoms as a genuine health concern (Dakic, Hay‐Smith, Lin, Cook, and Frawley 2023a). Exercising women also expressed a preference for being asked questions by a female health professional who was familiar to them (Dakic, Hay‐Smith, Lin, Cook, and Frawley 2023a). This may have increased the willingness of participants in the study to disclose symptoms. Limitations of the study include that participants completed the survey response in writing and there were a small number of responses that had to be removed due to illegible handwriting. It also limited auto‐logic available with online surveys. We were unable to categorise which PF symptoms had the greatest impact on performance. Our results were based on patient reported outcome measures and self‐report. We did not collect clinical measures to validate PF disorders.

## 5 Clinical Implications

We found that a broad range of PF symptoms were experienced by elite female rugby players, impacting performance for one in two players. Including pelvic health questions amongst routine general health and injury surveillance allowed for symptom identification in this cohort of rugby players. Early identification of PF symptoms allows for referral to those with specialist knowledge of PF assessment and management such as PF physiotherapists. Resources to understand and effectively manage symptoms can also be provided to athletes. Effective management of PF symptoms may reduce the impact on performance in elite female athletes without the need to reduce training intensity or restrict fluid intake.

## 6 Conclusion

Over 50% of elite female rugby players reported PF symptoms during sport. One in two symptomatic players reported a negative impact on their performance leading to training limitations, fluid intake reduction and effects on emotional well‐being. There is a need for targeted pelvic health screening, education and management strategies to reduce PF symptoms and their impact on performance in female rugby.

## Author Contributions

J.D., J.L. and S.S. contributed to the design of the study, generated aims and designed the protocol and ethics. S.S. was responsible for recruitment and data collection. J.D. conducted data analysis with assistance from E.H., S.C. and L.P. All authors were involved in interpretation of data. J.D. wrote first draft of the manuscript. All authors contributed to critical review of manuscript.

## Ethics Statement

The study was approved by a Human Ethics Research Committee (project number: 34414, 29/11/2023).

## Consent

Participants provided written consent for their data to be used in future research. Written consent to access the data was obtained from a representative at Rugby Australia.

## Conflicts of Interest

Dr Sharon Stay was a medical officer for Queensland Rugby Union. Dr. Jodie Dakic is a paid consultant advisor to the Hologic Women's Tennis Association Women's Health Taskforce and Labs.

## Data Availability

The data that support the findings of this study are available on request from the corresponding author. The data are not publicly available due to privacy or ethical restrictions.
